# MicroRNA miR-212-5p Regulates the MEK/ERK Signaling Pathway by Targeting A-Raf proto-oncogene serine/threonine-protein kinase (*ARAF*) to Regulate Cowshed PM_2.5_-Induced NR8383 Apoptosis

**DOI:** 10.3390/toxics11120981

**Published:** 2023-12-03

**Authors:** Ke Sun, Yize Sun, Yunna Jia, Xinran Duan, Zhenhua Ma, Xiqing Zhang, Lixia Wang, Yanbin Zhu, Yunhang Gao, Wangdui Basang

**Affiliations:** 1 College of Animal Science and Technology, Jilin Agricultural University, Changchun 130118, China; sun155198@163.com (K.S.);; 2 Institute of Animal Husbandry and Veterinary Medicine, Tibet Academy of Agricultural and Animal Husbandry Science, Lhasa 850009, China; 3 Northeast Institute of Geography and Agroecology, Chinese Academy of Sciences, Changchun 130102, China

**Keywords:** cowshed PM_2.5_, miR-212-5p, mitochondrial damage, MEK/ERK signaling pathway, apoptosis

## Abstract

**Objective:** To investigate the role of miR-212-5p-targeted *ARAF* during the apoptosis of rat alveolar macrophages induced by cowshed PM_2.5_. **Methods:** miRNA and related target genes and pathways were predicted using the KEGG, TargetScan, and other prediction websites. NR8383 macrophages were treated with cowshed PM_2.5_ to establish an in vitro lung injury model in rats; meanwhile, for the assessment of cell viability, apoptosis, intracellular calcium ions, and mitochondrial membrane potential in NR8383 cells, RT-qPCR was used to detect the expression of miR-212-5p and the target gene *ARAF*. **Results:** The bioinformatic analyses showed that miR-212-5p and *ARAF* were involved in PM_2.5_-associated cellular damage. Exposure to different concentrations (0 μg/mL, 60 μg/mL, 180 μg/mL, 300 μg/mL) with different durations (0 h, 12 h, 24 h, 48 h) of cowshed PM_2.5_ resulted in apoptosis, increased intracellular calcium ions, and decreased mitochondrial membrane potential. The miR-212-5p mimic group showed an up-regulation of Bax and cleaved Caspase 3 expression but decreased Bcl2 expression compared to the NC group, and overexpression of *ARAF* up-regulated the expression of p-MEK1/2 and p-ERK1/2 and simultaneously reversed the above phenomena. **Conclusions:** miR-212-5p targets *ARAF* to affect the cowshed PM_2.5_-induced apoptosis through the MEK/ERK signaling pathway, providing a potential target for relevant farming industry and pathology studies.

## 1. Introduction

PM_2.5_ has become one of the most important disease-causing factors today, with the World Health Organization (WHO) reporting that approximately 7 million people die every year from fine particulate pollution [1], which causes respiratory diseases [2]. PM_2.5_ has a complex composition that includes biocides, metal ions, aromatic hydrocarbons, and other disease-causing factors [3]. They spread where the air speed is high and pose a serious threat to the respiratory systems of humans and animals. Therefore, research on the pathophysiological mechanisms underlying the action of PM_2.5_ can provide a relevant entry point for PM_2.5_ prevention and control. Studies have shown that PM_2.5_ exposure can cause a variety of respiratory diseases, such as inflammation of airways and lung macrophages, pulmonary fibrosis, etc. [4,5,6]. At the same time, it may cause pyroptosis and ferroptosis, which can lead to pulmonary toxicity [7], particularly in livestock housing due to the dense and relatively enclosed animal population. The resulting fine particulate matter enters the lungs, causing loss of alveolar capillary membrane integrity, which in turn leads to lung destruction, fibrosing alveolitis, and lung tissue damage [8]. The immune system, especially macrophages, is a sensitive and powerful defense mechanism against harmful factors, including PM_2.5_ [9]. Normally, alveolar macrophages immediately remove PM_2.5_ through the phagocytosis–secretion–immunization network [10]. However, when persistent PM_2.5_ enters the respiratory system, macrophages in the lungs are hyper-activated, leading to an increase in acute damage [11]. Activated macrophages secrete pro-inflammatory cytokines (tumor necrosis factor alpha, interferons, and interleukins) and produce cytotoxic reactive oxygen species (ROS) [12,13], which in turn leads to apoptosis. Most of the PM_2.5_ in farm buildings comes from animal bedding, feed, and animal production activities, and may contain pathogens, leading to increased animal health problems, which will directly lead to a decline in economic efficiency. Thus, it becomes urgent to explore the virulence mechanism of PM_2.5_ in animal housing, which has great significance for the health and safety of animals and humans.

Mitochondrial apoptosis is the main pathway of endogenous apoptosis [14]. When it is stimulated by external stimuli (e.g., PM_2.5_), it causes a change in membrane potential which in turn leads to a change in mitochondrial membrane permeability and promotes apoptosis through the release and activation of apoptosis-related proteins including Bcl-2, Bax, Cyt-c, caspase 3, and caspase 9 [15,16].

The MAPK signaling pathway, which consists of RAS, RAF, MEK, and ERK, is involved in a variety of cellular response processes such as apoptosis, cell proliferation, and other processes [17]. There are three isoforms of RAF (*ARAF*, *BRAF*, and *CRAF*) that are phosphorylated by MEK [18]. The RAF family also has two pseudokinases, including a kinase inhibitor of Ras 1/2 (KSR1/2), which generally acts as a bridge between MEK and RAF and plays a key role in the activation of RAF [19]. Members of the RAF family bind to Ras-GTP and lead to its homo- and heterodimerization and kinase activation [20], which constitutes the first step in the kinase cascade. RAF phosphorylates and activates mitogen-activated protein kinases (MEKs), which in turn phosphorylates and activates extracellular signal-regulated protein kinases 1 and 2 (ERK1 and ERK2) [21]. Activating mutations in members of the RAF, MEK, and ERK families have all been identified in cancer, with BRAF mutations being by far the most common [22]. CRAF mutations also occur in human cancers, but less frequently in comparison with the RAF, MEK, and ERK families [23]. Both BRAF and CRAF encode active kinases, and knockdown of these kinases significantly reduces ERK signaling [24]. Kinase-independent functions of CRAF are associated with a variety of cellular phenotypes, including the regulation of mitochondria-dependent apoptosis by blocking the MST2/HIPPO pathway [25]. However, little has been reported in relation to *ARAF*, particularly in relation to *ARAF*-mediated MEK/ERK signaling pathways affecting mitochondrial dysfunction and thus apoptosis.

MicroRNAs (miRNAs) are non-coding RNAs that consist of 21–23 nucleotides and endogenously regulate cell development, proliferation, differentiation, and death by binding to the 3’ untranslated region (3’UTR) of target messenger RNA to promote their degradation [26]. As an important component of epigenetics, microRNAs might be valuable biomarkers of environmental exposure [27]. Particulate matter-induced alterations in microRNAs suggest their regulatory importance in PM-related diseases. Studies have shown that airborne particulate matter may contribute to bronchial inflammation and affect immune regulation in lung injury [28,29]. The expression of miRNAs is altered after PM_2.5_ exposure, and miR-4726-5p expression is elevated in response to PM_2.5_ stimulation and affects the development of coronary heart disease [30]. Exposure of H9C2 cells to PM_2.5_ results in the upregulation of miR-421 expression and downregulation of SIRT3 expression, triggering mitochondrial damage and consequent cardiovascular diseases [31]. Previous studies have shown that miR-212-5p is associated with the development of a variety of diseases, including ischemic brain injury [32], fat accumulation [33], nasopharyngeal carcinoma, and various cancers such as breast cancer [34]. However, there are limited reports on the mechanism of miR-212-5p’s regulation of apoptosis in lung injury under cowshed PM_2.5_ stimulation. Thus, we proposed a scientific hypothesis that miR-212-5p-targeted *ARAF* plays an important role in the mechanism of rat alveolar macrophage apoptosis induced by cowshed PM_2.5_ through the MEK/ERK signaling pathway.

In this study, using the in vivo rat infection model constructed by our group, we found that miR-212-5p was differentially expressed based on transcriptome data analysis [35], and ARAF was also predicted as its target gene through bioinformatic analyses. We investigated the mechanism of miR-212-5p-targeted *ARAF* in PM_2.5_-induced apoptosis of rat alveolar macrophages in vitro, aiming to provide gene therapy targets and a scientific basis for PM_2.5_-induced apoptosis triggering respiratory diseases at the level of miRNA regulation.

## 2. Materials and Methods

### 2.1. Chemicals and Reagents

DMSO (C2H6OS, CAS: 67–68–5) was obtained from Sigma-Aldrich, Merck, Germany, while anti-MEK1/2 antibody (9G3) (sc-81504), anti-ERK1/2 antibody (C-9) (sc-514302), anti-Bax antibody (sc-7480), and anti-caspase 3 antibody (sc-56053) were obtained from Santa Cruz Biotechnology, USA. Anti-p-MEK1/2 (S217/221) antibody (9154T) was obtained from CST, USA, while anti-p-ERK1/2 antibody (AP0472) and anti-*ARAF* antibody (A16346) were obtained from ABclonal, China. Anti-Bcl-2 antibody (26593-1-AP), anti-cytochrome C antibody (66264-1-lg), anti-GAPDH antibody (60004-1-Ig), anti-β-actin antibody (20536-1-AP), and secondary goat anti-rabbit and rabbit anti-mouse antibodies were purchased from Proteintech Group (Wuhan, China). Anti-caspase 9 antibody (WL03421) was obtained from Wanleibio, China.

### 2.2. PM_2.5_ Sample Collection

PM_2.5_ was collected using a multi-level flow particulate sampler (2030, Laoying Haina Photovoltaic Environmental Protection Group Co., Qingdao, China) from a dairy cattle farm in Changchun, Jilin Province [36,37,38]. The sampler was located at the center of the cow farm, 1.5 m above the ground, with a sampling flow rate of 100 L/min that operated continuously for 24 h. After collection, ultra-pure water without endotoxin was used for elution, which was then filtered through six layers of sterile gauze and freeze-dried using a vacuum lyophilizer. The final PM_2.5_ was made into a master batch of 1 × 10^4^ μg/mL and preserved. Ultrasound was used to shake it well before each use to prevent it from caking into larger particles.

### 2.3. Cell Culture

Rat alveolar macrophage cell line (NR8383) and human embryonic kidney cell line (HEK-293T) were purchased from Shanghai Cell Bank, China, and cultured in DMEM medium (GIBCO, CA, USA) with 10% FBS (fetal bovine serum, GIBCO, CA, USA) and 1% penicillin–streptomycin (C0222, Beyotime Biotechnology, Shanghai, China). The cells were grown within a humidified 5% CO_2_ atmosphere at 37 °C.

### 2.4. CCK8 Cell Viability Assay

We use the CCK-8 kit to detect cell viability (C0037, Beyotime Biotechnology, China). NR8383 cells were inoculated into a 96-well plates at a density of 5 × 10^3^ cells per well. PM_2.5_ exposure treatments were performed in the cowshed using concentrations of 0, 60, 180, and 300 μg/mL, with 5 biological replicates for each concentration. A 10 μL volume of the CCK-8 working solution was added to each well and incubated for 1 h, then the absorbance value at 450 nm was detected using a Microplate Reader (Thermo Fisher, Waltham, MA USA). Cell viability was measured according to the manufacturer’s instructions.

### 2.5. Plasmid Construction and Cell Transfection

We transfected miR-212-5p mimics and miR-212-5p inhibitor into NR8383 cells to construct miR-212-5p overexpression and silencing cell lines, respectively. miRNA NC and miRNA inhibitor NC were the corresponding negative controls. For the overexpression of *ARAF*, we linked the complete CDS sequence of the *ARAF* gene to the pcDNA3.1 plasmid to construct the *ARAF* overexpression vector, with the blank pcDNA3.1 plasmid as the negative control. The miR-212-5p mimics, miR-212-5p inhibitor, miRNA NC, miRNA inhibitor NC, and pcDNA3.1 were purchased from GenePharma (Suzhou China). NR8383 cells were seeded into 6-well plates and cultured until about 60% confluence. The Lipofectamine 3000 Kit (Thermo Fisher, USA) was used to transfect 50 nM oligonucleotides or 2 μg plasmids into the NR8383 cells.

### 2.6. Determination of Calcium Ion Concentration

We used the Fluo-4 AM calcium fluorescent probe method to determine intracellular calcium levels according to the manufacturer’s protocol (S1061S, Beyotime Biotechnology, China). NR8383 cells were first inoculated into cell culture plates, the medium was aspirated after reaching a density of 10^6^, and the cells were washed with PBS. Then, 500 μL of Fluo-4 working solution was added for staining and incubated at 37 °C for 30 min in the dark, after which, the cells were examined using a fluorescence microscope, and then the data were analyzed using Image J software 1.8.0.

### 2.7. Determination of Reactive Oxygen Species (ROS) Level

The intracellular ROS level was determined using a fluorescent probe: dichloro-dihydro-fluorescein diacetate (DCFH-DA). The cells were first inoculated in cell culture plates overnight at a density of 1 × 10^5^/mL according to the manufacturer’s protocol (S0033S, Beyotime Biotechnology, China). The cells were then observed for their growth status, the cell culture medium was aspirated, and 1 mL of diluted prepared DCFH-DA working solution was added to each well. The cells were then incubated in a humidified 5% CO_2_ atmosphere at 37 °C for 20 min. The cells were washed three times with phosphate-buffered saline (PBS) and then collected, and their luminescence intensity was measured using a fluorescent microscope (Olympus, Tokyo, Japan). The experiment was repeated three times for each sample. Finally, the analysis was performed using Image Pro Plus software 6.0 (Media Contronetics, Rockville, MD, USA).

### 2.8. JC-1 Detection of Mitochondrial Membrane Potential

We used the Enhanced Mitochondrial Membrane Potential Assay Kit (JC-1) to determine the mitochondrial membrane potential (Δψm) (C2003S, Beyotime Biotechnology, China). We suspended 5 × 10^5^ cells in the cell culture solution, added a 500 μL JC-1 staining working solution, and incubated them at 37 °C for 20 min in a cell culture incubator. After the incubation was completed, the cells were collected, and the JC-1 dyeing buffer was washed twice. After being re-suspended, a fluorescent microplate reader (Spark, Tecan Group Ltd., Männedorf, Switzerland) and fluorescent microscope (Olympus, Tokyo, Japan) were used to measure the staining.

### 2.9. Real-Time Quantitative PCR

Total RNA was extracted from cells using a column total RNA extraction kit (B511321, Sangon Biotech, Shanghai, China) and reverse transcription into cDNA was performed using a PrimeScriptTM RT kit (RR047A, TaKaRa, Dalian, China). The primers for miR-212-5p, *ARAF*, U6, and GAPDH listed in Table 1 were obtained from Sangon Biotech, Shanghai, China. RT-qPCR was performed according to the manufacturer’s protocol for the RT-qPCR kit (RR820A, TaKaRa, Dalian, China). The relative expression of genes was calculated using the 2^−ΔΔCt^ method.

### 2.10. Dual-Luciferase Reporter Assay

The putative binding sites for miR-212-5p and *ARAF* were predicted using the online software TargetScan7.2 (TargetScanHuman 7.2). The wild-type *ARAF* (*ARAF*-WT) and mutant *ARAF* (*ARAF*-MUT) sequences were amplified, and their binding sites to rat miR-212-5p were cloned into the pmir-GLO dual-luciferase reporter vector. The constructed *ARAF* vector was co-transfected with NC or miR-212-5p in HEK-293T cells since the transfection efficiency of HEK-293T is higher than that of NR8383 cells. After 48 h of transfection, the luciferase activity was measured and normalized using a Dual-Luciferase Reporter Kit (DL101, Vazyme, Nanjing, China).

### 2.11. Flow Cytometry

The apoptosis analysis was performed using the Annexin V-FITC/PI kit (556547, BD PharmingenTM, Franklin Lakes, NJ, USA). NR8383 cells were collected after 72 h of transfection (1 × 10^6^ cells/time), washed with cold PBS, suspended in 1 mL of 1 × Binding Buffer, centrifuged at 300 rpm for 10 min, and the supernatant was discarded. The cells were resuspended in 1 mL of 1 × Binding Buffer to a density of 1 × 10^6^ cells/mL. A 100 μL volume of cells suspension (1 × 10^5^ cells) was incubated with 5 μL of Annexin V-FITC and 5 μL of PI for 15 min at room temperature in a dark environment. A 500 μL volume of PBS was added and gently mixed. The apoptotic cells were finally examined using a BD LSRFortessa (BD Biosciences, Franklin Lakes, NJ, USA).

### 2.12. Western Blotting

Briefly, we lysed the cultured cells using RIPA lysis solution containing 1% PMSF which were then centrifuged and the supernatant was taken after 30–40 min at 4 °C. The extracted protein concentrations were determined using the BCA protein quantification method and then SDS-PAGE gel electrophoresis was performed, the proteins were transferred to a PVDV membrane and then blocked using a blocking solution (5% skimmed milk powder). Then, the membranes were incubated overnight at 4 °C with the corresponding primary antibody and an HRP-conjugated secondary antibody (anti-mouse/rabbit antibody) for 1 h at room temperature and finally ECL luminescent solution was added to detect the protein bands using an Amersham Imager 680 (GE Healthcare, Sunnyvale, Chicago, Illinois, USA). The image analysis was performed using Image J.

### 2.13. Statistical Analysis

The experiments were repeated at least three times, and the values are shown as the mean ± SE of the three measurements. GraphPad Prism version 8.0 software (GraphPad Software, San Diego, CA, USA) was used to plot the experimental results. Student’s *t*-tests were used to evaluate the data. Relative gene expression data were calculated using the 2^−ΔΔCT^ method. Differences between the groups were analyzed by one-way analysis of variance (ANOVA), followed by Duncan’s multiple comparison tests. A *p* value less than 0.05 was considered statistically significant.

## 3. Results

### 3.1. PM_2.5_ Causes Apoptosis in Rat NR8383 Cells

**Figure 1:**
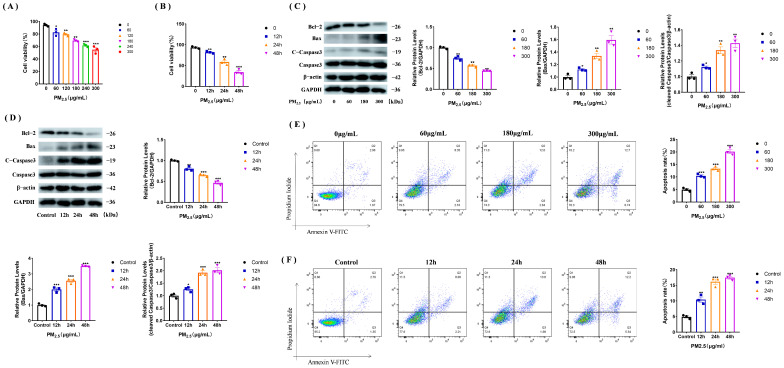
The cowshed PM_2.5_ reduces NR8383 cell viability and promotes apoptosis. (**A**) CCK8 assay for cell viability of the cells stimulated with cowshed PM_2.5_ (0, 60, 120, 180, 240 and 300 μg/mL) for 12 h assayed. (**B**) Cells were stimulated using 180 μg/mL of the cowshed PM_2.5_ for 0, 12, 24, and 48 h and then cell viability was assayed using the CCK8 assay. (**C**,**D**) Expression of apoptosis-related proteins after stimulation of NR8383 cells at different concentrations of the cowshed PM_2.5_ (**C**) at different time points (**D**). (**E**,**F**) Apoptosis analysis by flow cytometry using Annexin V-FITC and PI staining of NR8383 cells after stimulation with the cowshed PM_2.5_ at different concentrations (**E**) at different time points (**F**). All groups were analyzed in comparison to 0 μg/mL or control group. Each experiment was repeated at least three times, and values are shown as the mean ± SE of the three measurements. * *p* < 0.05, ** *p* < 0.01, *** *p* < 0.001.

We used the Cell Counting Kit-8 (CCK-8) to detect the cell viability of rat NR8383 cells stimulated with the cowshed PM_2.5_ at different concentrations (0, 60, 120, 180, 240, and 300 μg/mL) for 12 h. The results showed that the cell viability decreased with increasing concentrations of PM_2.5_ (Figure 1A). Based on these results, we chose 60, 180, and 300 μg/mL as the low, medium, and high concentrations for subsequent use. The cell viability reached 70% after the 180 μg/mL stimulation, which was chosen as the single stimulus amount to detect the cell viability at different time points after cowshed PM_2.5_ stimulation (0, 12, 24, and 48 h) (Figure 1B). The results showed that cell viability decreased in a time-dependent manner. We have also examined the expression of apoptosis-related proteins, and found that Bcl-2 levels decreased, but the expression of Bax and caspase 3 increased. PM_2.5_ also decreased cell viability and induced apoptosis with increasing the concentration and time (Figure 1C,D). Subsequently, we examined apoptosis using flow cytometry and showed that PM_2.5_ caused apoptosis in a time- and concentration-dependent manner (Figure 1E,F). The results suggest that PM_2.5_ reduces cell viability, leading to apoptosis.

### 3.2. PM_2.5_ Increases Calcium Ions and ROS to Trigger Mitochondrial Damage

**Figure 2:**
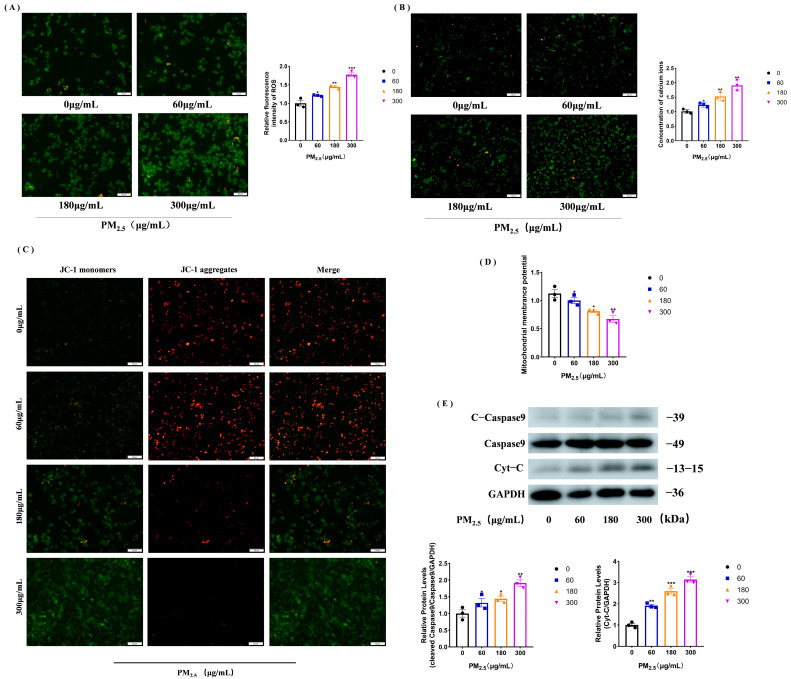
The cowshed PM_2.5_ increases calcium ion and ROS levels and thus triggers mitochondrial damage. (**A**) Changes in calcium ion concentration using fluorescence microscopy after the addition of Fluo-4 AM fluorescent probe to cells stimulated with different concentrations of the cowshed PM_2.5_. (**B**) Changes in ROS were observed using fluorescence microscopy after the addition of DCFH-DA fluorescent probe to cells stimulated with different concentrations of the cowshed PM_2.5_. (**C**,**D**) Cells stimulated with different concentrations of the cowshed PM_2.5_ were incubated with the JC-1 fluorescent probe and fluorescence microscopy was used to observe the changes in mitochondrial membrane potential (**C**). Simultaneous detection of changes in membrane potential using a fluorescent microplate reader (**D**). (**E**) Mitochondrial damage-associated protein assay was performed on cells after the treatment. All groups were analyzed in comparison to 0 μg/mL. Each experiment was repeated at least three times, and values are shown as the mean ± SE of the three measurements. * *p* < 0.05, ** *p* < 0.01, *** *p* < 0.001.

We used a fluoroprobe to detect calcium ion levels in NR8383 cells after stimulation with different concentrations of the cowshed PM_2.5_ and found that the calcium ion concentration increased in a cowshed PM_2.5_ concentration-dependent manner (Figure 2A). Similarly, the results of the luciferase labeling experiment showed that after PM_2.5_ stimulation, the ROS level increased and was proportional to the PM_2.5_ concentration (Figure 2B). Using the fluorescent probe JC-1, it was found that the green fluorescence gradually intensified and the red fluorescence decreased after PM_2.5_ treatment compared to the control, indicating that the PM_2.5_ treatment resulted in an increase in monomer and a decrease in polymer levels, resulting in a decrease in mitochondrial membrane potential, which was again corroborated by the luciferase labeling experiment (Figure 2C,D). We then examined the Cyt-c and caspase 9 protein levels and found that the expression of both proteins also increased with the cowshed PM_2.5_ treatment (Figure 2E). These results suggest that the cowshed PM_2.5_ can promote intracellular calcium and ROS production, reduce the mitochondrial membrane potential, and increase the expression of related proteins, thereby triggering mitochondrial damage.

### 3.3. miR-212-5p Expression Increases under the Cowshed PM_2.5_ Stimulation

**Figure 3:**
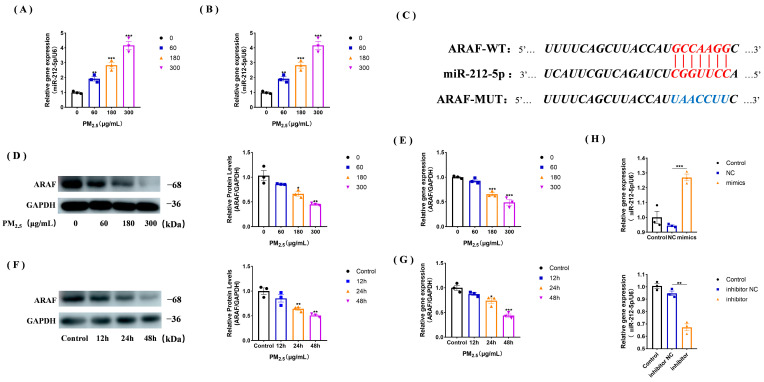
miR-212-5p expression increases after PM_2.5_ stimulation and its binds to *ARAF*. (**A**,**B**) qPCR validation of miR-212-5p expression changes after the cowshed PM_2.5_ cells stimulation at different concentrations (**A**) and different durations (**B**). (**C**) The prediction website identified a possible binding relationship between miR-212-5p and *ARAF*. (**D**,**E**) *ARAF* expression at the protein (**D**) and mRNA (**E**) levels after cells were stimulated with different concentrations of the cowshed PM_2.5_. (**F**,**G**) The expression of *ARAF* decreased at the protein (**F**) and mRNA (**G**) levels after cells were stimulated with the cowshed PM_2.5_ for different durations. (**H**) Expression of miR-212-5p after transfection with mimics and inhibitors. All groups were analyzed in comparison to 0 ng/μL or control group. Each experiment was repeated at least three times, and values are shown as the mean ± SE of the three measurements. * *p* < 0.05, ** *p* < 0.01, *** *p* < 0.001.

miR-212-5p was found to be differentially expressed from the transcriptomic data (Table S1) and, given its multiple biological functions, we conjectured that it might have some role in cowshed PM_2.5_ stimulation. Therefore, we examined miR-212-5p expression after stimulating NR8383 cells with concentrations of the cowshed PM_2.5_ for different durations, and the results showed that miR-212-5p was up-regulated in a time- and cowshed PM_2.5_ concentration-dependent manner (Figure 3A,B).

### 3.4. ARAF Is a Potential Target of miR-212-5p and Its Expression Is Down-Regulated in the Cowshed PM_2.5_-Stimulated NR8383 Cells

*ARAF* was predicted to be an miR-212-5p target gene using TargetScan, miRDB, and other websites. At the same time, we found that miR-212-5p may bind to the 3’UTR region of *ARAF* (Figure 3C). Subsequently, we verified by RT-qPCR and Western blot that *ARAF* expression was down-regulated after cowshed PM_2.5_ stimulation (Figure 3D–G). Next, we transfected miR-212-5p mimics and inhibitors to verify the transfection efficiency, and the results showed successful overexpression and knockdown of miR-212-5p (Figure 3H).

### 3.5. miR-212-5p Targets ARAF, Promoting Mitochondrial Injury and Apoptosis after Cowshed PM_2.5_ Stimulation

**Figure 4:**
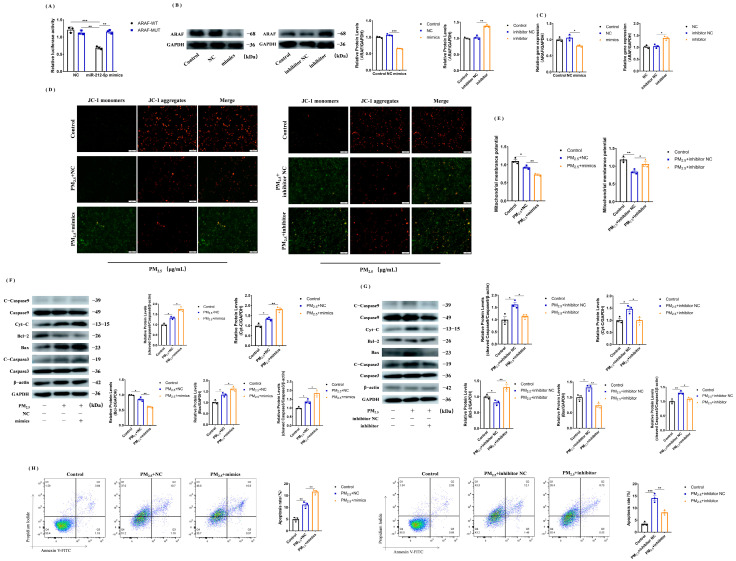
miR-212-5p binds to *ARAF* and promotes mitochondrial damage and apoptosis in response to PM_2.5_ stimulation. (**A**) Dual-luciferase reporter assay verified that miR-212-5p can target and bind to *ARAF*. (**B**,**C**) Western blotting (**B**) and qPCR (**C**) verified that miR-212-5p and *ARAF* are both negatively correlated. (**D**) Detection of mitochondrial membrane potential using the JC-1 probe. (**E**) Detection of mitochondrial membrane potential using a fluorescent microplate reader. (**F**,**G**) Changes in mitochondrial damage and apoptosis-related protein expression after transfection with miR-212-5p mimics (**F**) and inhibitor (**G**) in the cowshed PM_2.5_-stimulated cells. (**H**) Apoptosis rates after transfection with mimics or inhibitor were detected using flow cytometry. All groups were analyzed in comparison with the control or NC groups. Each experiment was repeated at least three times, and values are shown as the mean ± SE of the three measurements. * *p* < 0.05, ** *p* < 0.01, *** *p* < 0.001.

To demonstrate that *ARAF* is a true target of miR-212-5p, the 3’UTR of the rat *ARAF* gene was introduced into the pmir-GLO reporter plasmid, and subsequent experiments using a dual-luciferase reporter assay revealed a reduction in dual-luciferase activity by miR-212-5p compared to the control, while *ARAF-MUT* showed no significant changes (Figure 4A). To demonstrate the regulatory relationship between miR-212-5p and *ARAF*, cells transfected with NC, mimics, inhibitor NC, and inhibitor were assayed for *ARAF* expression by Western blotting and RT-qPCR (Figure 4B,C). The results showed that *ARAF* expression was reduced upon overexpression of miR-212-5p, and the two were negatively correlated. To verify the role of miR-212-5p in cells, after transfection of the mimics, inhibitor, and their negative controls, the mitochondrial membrane potential and apoptosis-related proteins were analyzed after cowshed PM_2.5_ stimulation (Figure 4D–G). The results showed that after overexpression of miR-212-5p, the mitochondrial membrane potential was decreased, and the expression of Cyt-c, caspase 9, caspase 3, and Bax increased while the expression of Bcl-2 decreased. The opposite results were achieved with the addition of the inhibitor. Flow cytometry also confirmed the above conclusions (Figure 4H). In conclusion, we can say that overexpression of miR-212-5p leads to mitochondrial damage and promotes apoptosis.

### 3.6. Activation of MEK/ERK Signaling Pathway after Overexpression of ARAF Inhibits the Cowshed PM_2.5_-Induced Apoptosis

**Figure 5:**
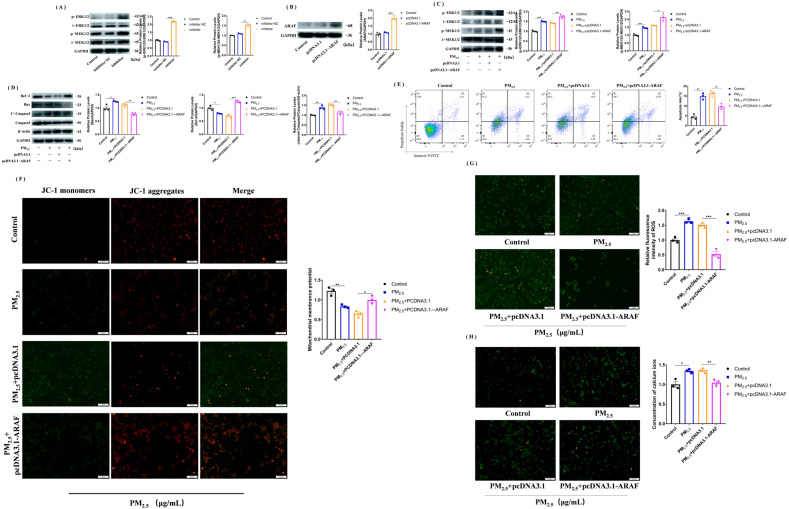
Overexpression of *ARAF* activates the MEK/ERK signaling pathway to affect apoptosis. (**A**) Changes in p-MEK1/2 and p-ERK1/2 protein expression after transfection with miR-212-5p inhibitor. (**B**) Western blotting verified the successful construction of pcDNA3.1-*ARAF*. (**C**) Changes in p-MEK1/2 and p-ERK1/2 protein expression after transfection of pcDNA3.1-*ARAF* in response to the cowshed PM_2.5_ stimulation. (**D**) Detection of associated proteins after overexpression of *ARAF*. (**E**) Detection of apoptosis rate after overexpression of *ARAF* using flow cytometry. (**F**–**H**) Mitochondrial membrane potential (**F**), ROS (**G**), and calcium (**H**) changes after overexpression of *ARAF*. All groups were analyzed in comparison with control or pcDNA3.1 empty vector groups. Each experiment was repeated at least three times, and values are shown as the mean ± SE of the three measurements. * *p* < 0.05, ** *p* < 0.01, *** *p* < 0.001.

Previously, it was verified that miR-212-5p could promote apoptosis, but whether it was related to the MEK/ERK pathway is not known. We found that p-MEK1/2 and p-ERK1/2 expression was upregulated after transfection with the miR-212-5p inhibitor (Figure 5A). To verify the specific apoptotic mechanisms within the cells, we constructed pcDNA3.1-*ARAF* and transfected it into cells, and found that p-MEK1/2 and p-ERK1/2 expression was up-regulated in the presence of the cowshed PM_2.5_ (Figure 5B,C), indicating that the MEK/ERK signaling pathway was activated. We then examined the apoptotic proteins and mitochondria-associated proteins and found that as the expression of *ARAF* increased, Bcl-2 expression was up-regulated and Bax and caspase 3 expression was down-regulated, which was similarly confirmed by flow cytometry (Figure 5D,E). Similarly, after overexpression of *ARAF*, the mitochondrial membrane potential, ROS level, and calcium profile were all reversed (Figure 5F–H). The results suggest that overexpression of *ARAF* can inhibit the onset of apoptosis by activating the MEK/ERK signaling pathway.

## 4. Discussion

To date, the number of deaths per year from airborne particulate matter has increased dramatically, while the more complex air environment at the farms poses a serious threat to human and animal health. The literature suggests that particulate matter emissions are higher in livestock and poultry housing, particularly in cowsheds, than in the general area [39,40]. This complex environment increases the expression of pro-inflammatory factors, which ultimately contribute to a variety of diseases, including non-small cell lung cancer (NSCLC) and cerebral atherosclerosis, which is consistent with our hypothesis [41,42]. This has a direct impact on the economic performance of farms and has a deterrent effect on economic development. Compared with other farms, cattle farms derive their economic benefits from meat and milk production, which occupy a large share of the market, and the monitoring of the health of the animals themselves should be taken into account. Therefore, it is particularly important to explore the specific intracellular mechanisms of cowshed PM_2.5_ regulation.

Macrophages are the first barrier against invaders. When a bacterium or virus enters the organism, macrophages quickly recognize the foreign invader. They form a small pocket in their outer cell membrane to encapsulate the invader or to digest and destroy them. At the same time, macrophages will call on T-cells and B-cells to work together to fight the infection. Previously, our group constructed an in vivo rat infection model and performed transcriptome sequencing. In this study, we constructed an in vitro injury model by treating rat alveolar macrophages with cowshed PM_2.5_, with the aim of discovering a new mechanism of lung injury by cowshed PM_2.5_. The PM_2.5_ treatment reduced the expression of *ARAF* in NR8383 cells while inducing alterations in the miRNA profile of rat lungs, consistent with the previous reports [43]. Notably, among the group of dysregulated miRNAs, miR-212-5p increased in response to bovine housing PM_2.5_ and promoted apoptosis in NR8383 cells through the downregulation of *ARAF* expression.

The RAF kinase family has been reported to be involved in the process of tumor progression, especially BRAF and CRAF, and their mutations lead to a number of outcomes, such as tumor metastasis and apoptosis [44]. However, mutations in *ARAF* have been rarely reported, which is why the aim of this study was to discuss the specific role and regulatory mechanism that *ARAF* plays in the cowshed PM_2.5_-induced apoptosis.

A large number of miRNAs can regulate various physiological activities in organisms. The literature shows that there are a variety of miRNAs that are abnormally expressed in response to airborne particulate exposure, while PM_2.5_ has a more serious harmful effect on organisms [45]. This has become strong epiphenomenal evidence in this article to understand the process of apoptosis caused by cowshed PM_2.5_. It is undeniable that miR-212-5p regulates multiple target genes, which have the same or different functions [32,46,47], all of which suggest that miR-212-5p has complex biological functions depending on the target genes and cellular context. Some articles have reported that miR-212-5p can regulate some physiological activities, such as cell proliferation and epithelial mesenchymal transition [48,49], but the regulation of apoptosis after cowshed PM_2.5_ stimulation by miR-212-5p has not been reported.

In our experiments, we concluded that the expression of miR-212-5p was altered in response to the cowshed PM_2.5_ stimulation. Interestingly, we found from RT-qPCR that the expression of miR-212-5p was significantly increased after 24 h of the cowshed PM_2.5_ stimulation, which corresponded to a significant increase in apoptosis 24 and 48 h after the cowshed PM_2.5_ exposure. It is reasonable to suspect that the change in miR-212-5p expression may have occurred in the pre-apoptotic phase and that the change in miR-212-5p expression led to an increase in the rate of apoptosis. Thus, miR-212-5p may regulate many physiological functions in cells, including apoptosis. In this article, we mainly discussed apoptosis levels at the cellular level in rats after stimulation with cowsheds PM_2.5_. In the next step, we will focus on cells from other species, such as human macrophages or epithelial cells, to further explore whether this mechanism is feasible, which will have significant implications for the health and safety of breeders. In addition, it is unknown whether there were other related results in the programmed death mode, and it is necessary to explore the toxic mechanism of the cowsheds PM_2.5_ and will also be the focus of our next research.

In the last section, we first verified that the miR-212-5p inhibitor may activate the MEK/ERK signaling pathway, and secondly, we verified that the overexpression of *ARAF* also activates the MEK/ERK signaling pathway when stimulated by the cowshed PM_2.5_. However, it is worth noting that, compared with the control group, the cowshed PM_2.5_ group also had elevated expression of p-MEK1/2 and p-ERK1/2 proteins, which may be due to the fact that when the cells were stimulated by the cowshed PM_2.5_ and apoptosis occurred, it triggered the feedback regulation, which led to the elevation of the expression of p-MEK1/2 and p-ERK1/2. Moreover, due to the complexity of the pro-apoptotic network of the cowshed PM_2.5_, it is possible that other pathways are also playing an important role in influencing the results. However, the overexpression group had a higher expression of p-MEK1/2 and p-ERK1/2, which had more obvious effects on pathway activation.

In this article, we first found that cell viability decreased after cowshed PM_2.5_ stimulation in a time- and concentration-dependent manner, which is consistent with previous studies [50]. It has been suggested in the literature that intracellular reactive oxygen species and calcium ions may contribute to lung injury [51,52]. We then confirmed our suspicions experimentally and found that intracellular reactive oxygen species and calcium ion levels increased significantly after the cowshed PM_2.5_ stimulation, which might be one of the reasons for the induction of apoptosis. As the mitochondrial apoptotic pathway is one of the main pathways through which apoptosis occurs [53], the accumulation of reactive oxygen species led us to associate it with the mitochondrial apoptotic pathway [54], as evidenced by the decrease in mitochondrial membrane potential and proteins such as cytochrome C, which is also consistent with previous experiments. Subsequently, a change in expression of miR-212-5p was found, and its target gene *ARAF* was confirmed using a dual-luciferase assay. The KEGG pathway database and the construction of overexpression vectors revealed that the MEK/ERK signaling pathway was specifically activated and led to the expression of anti-apoptotic proteins that inhibited the expression of pro-apoptotic proteins. In conclusion, our new findings suggest that the miR-212-5p/*ARAF*/MEK-ERK axis is involved in the cowshed PM_2.5_-induced apoptosis in the alveolar macrophage cell line (NR8383).

## 5. Conclusions

In conclusion, our study showed that in the cowshed PM_2.5_-induced apoptosis in NR8383 cells, the resistance to apoptosis occurs through overexpression of *ARAF* and activation of the MEK/ERK signaling pathway. These effects could be regulated by miR-212-5p. Therefore, we found that the cowshed PM_2.5_ affects mitochondrial damage in NR8383 cells through the miR-212-5p/*ARAF*/MEK-ERK axis and thus promotes apoptosis. miR-212-5p is upregulated as a pro-apoptotic regulator in the cowshed PM_2.5_-treated NR8383 cells and inhibits the protective effect of *ARAF* on cells. Our results demonstrate a novel mechanism of the miR-212-5p/*ARAF*/MEK-ERK axis in the cowshed PM_2.5_-induced apoptosis, providing a new perspective for the study of the cowshed PM_2.5_-induced lung toxicity.

## Figures and Tables

**Table 1:** The primers for qRT-PCR.

Gene	Primer Sequence (5′-3′)
*ARAF*	Forward: CAGTGAGGTACAGCTGTTGAAG
	Reverse: GCCACCTTGAGCACTTTCA
*GAPDH*	Forward: CCTGCACCACCAACTGCTTA
	Reverse: CATCACGCCACAGCTTCCA
U6	Forward: CTCGCTTCGGCAGCACA
	Reverse: AACGCTTCACGAATTTGCGT
miR-212-5p	Forward: CGCGACCTTGGCTCTAGACTG
	Reverse: AGTGCAGGGTCCGAGGTATT

## Data Availability

The data presented in this study are available on request from the corresponding author.

## Supplementary Materials

The following supporting information can be downloaded at: https://www.mdpi.com/article/10.3390/toxics11120981/s1, Table S1: Differentially expressed miRNAs.
