# Supplementary material for: MicroRNA miR-212-5p Regulates the MEK/ERK Signaling Pathway by Targeting A-Raf proto-oncogene serine/threonine-protein kinase (ARAF) to Regulate Cowshed PM2.5-Induced NR8383 Apoptosis
Source: Toxics. 2023 Dec 3;11(12):981. doi: 10.3390/toxics11120981 (PMC10748134; doi:10.3390/toxics11120981)
Supplement: Supplementary file 1 [file toxics-11-00981-s001.zip › Western blot raw date.pdf]

Figure1-C

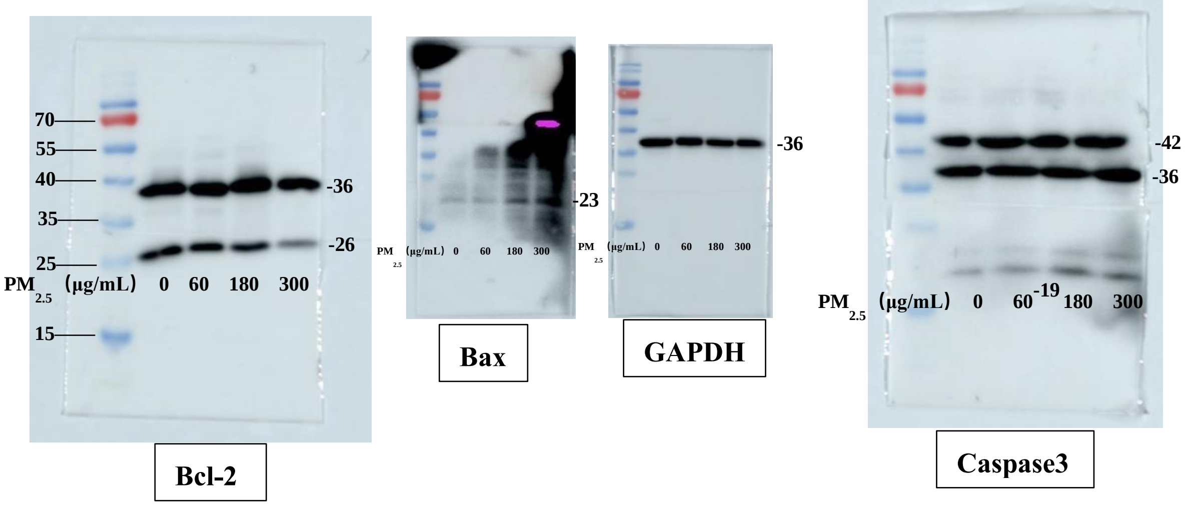

Figure1-D

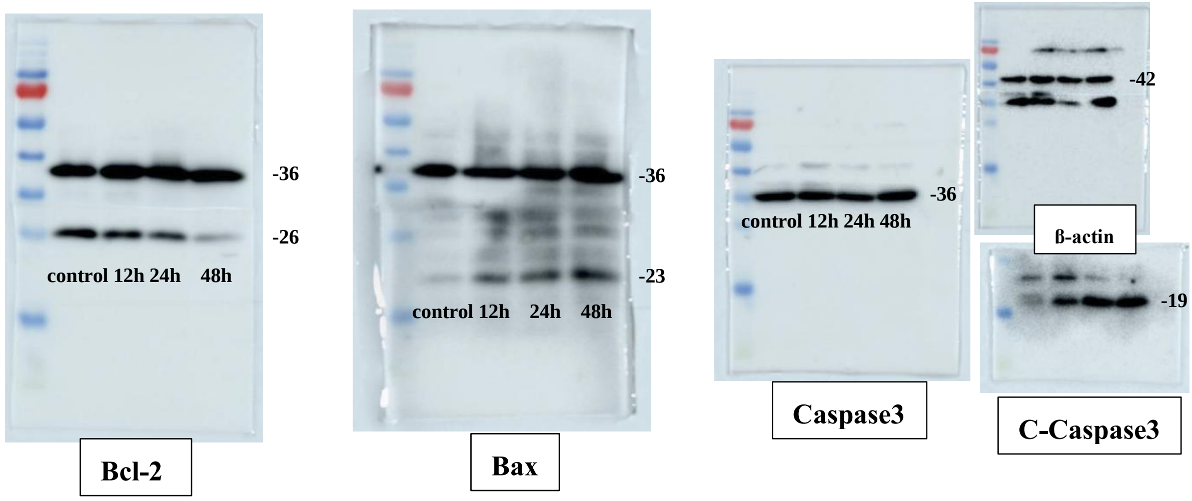

Figure2-E

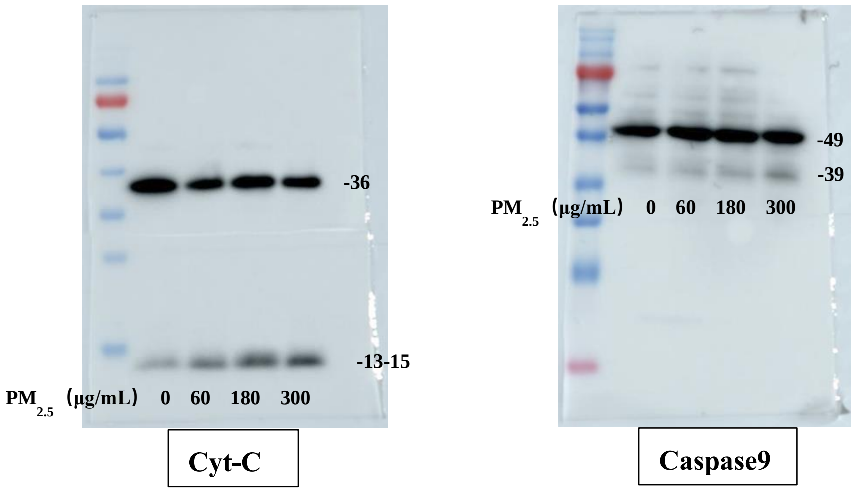

Figure3-F、G

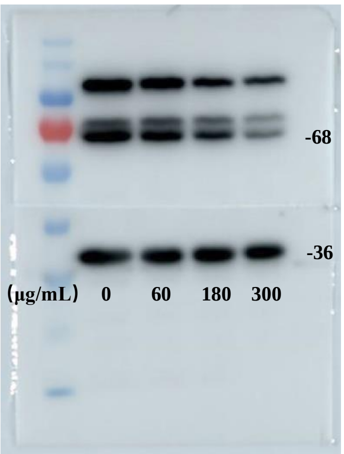

ARAF

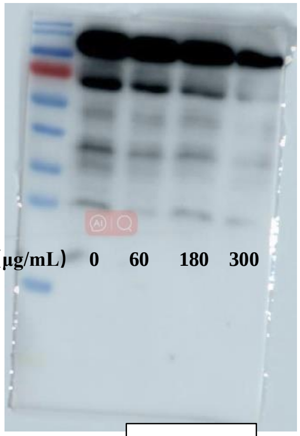

ARAF

Figure4-B

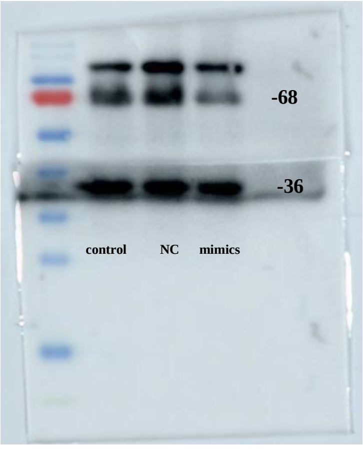

ARAF

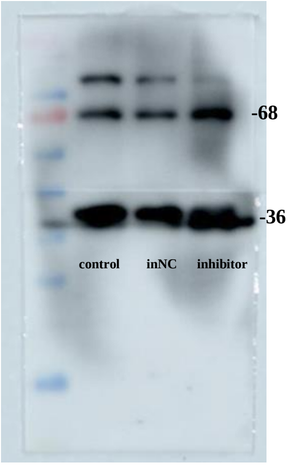

ARAF

Figure4-F

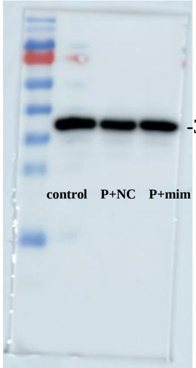

GAPDH

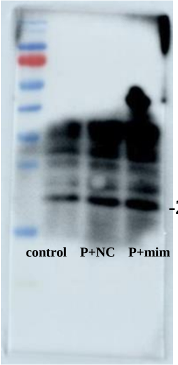

Bax

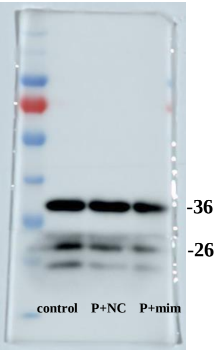

Bcl-2

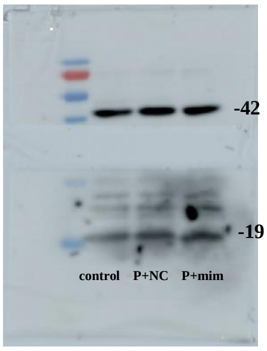

Caspase3

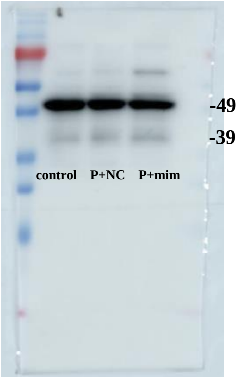

Caspase9

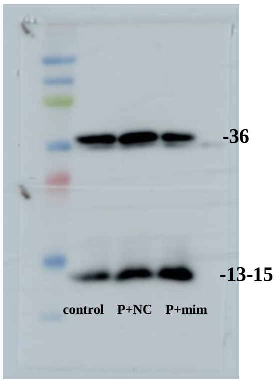

Cyt-c

Figure4-G

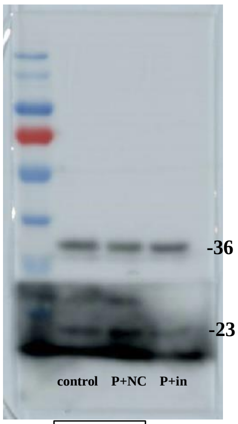

Bax

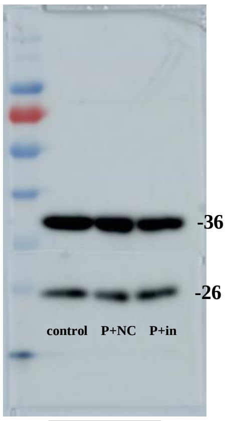

Bcl-2

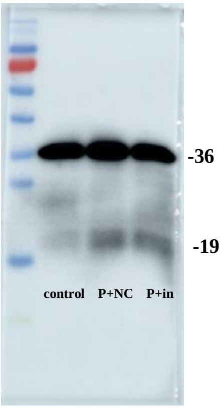

Caspase-3

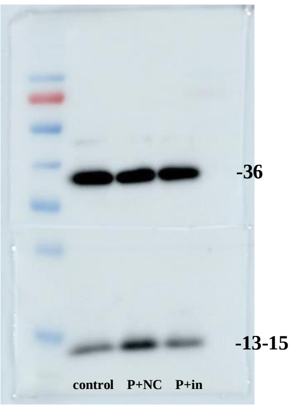

Cyt-c

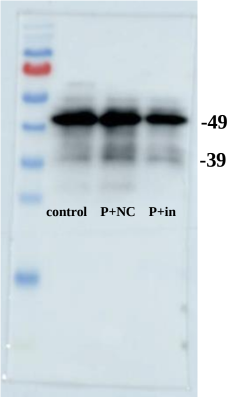

Caspase9

Figure5-A

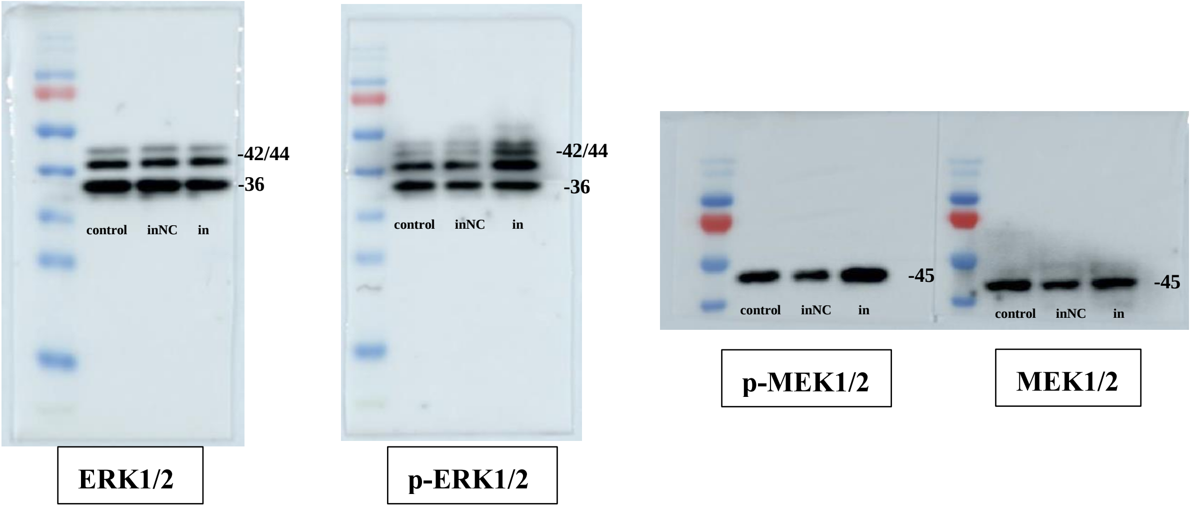

Figure5-B

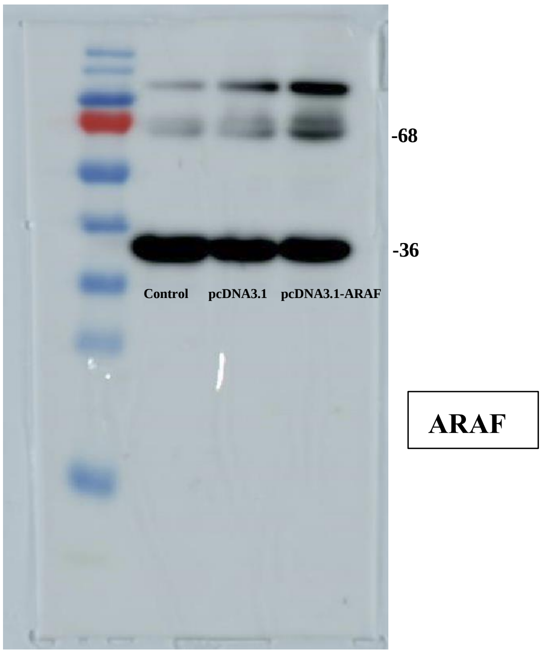

Figure5-C

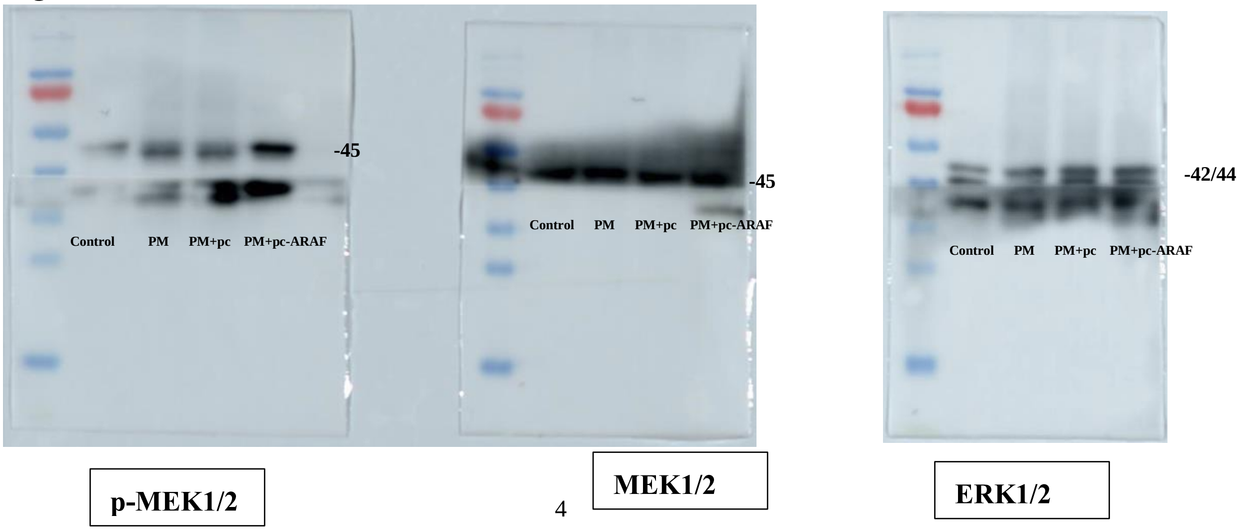

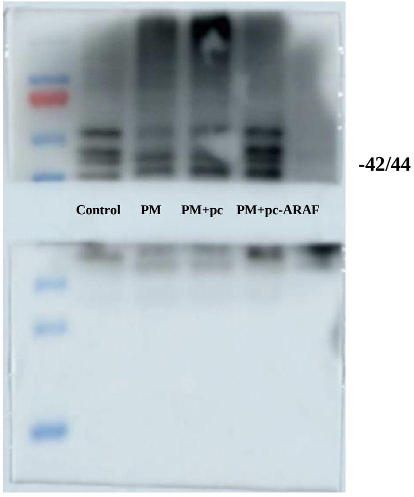

p-ERK1/2

Figure5-D

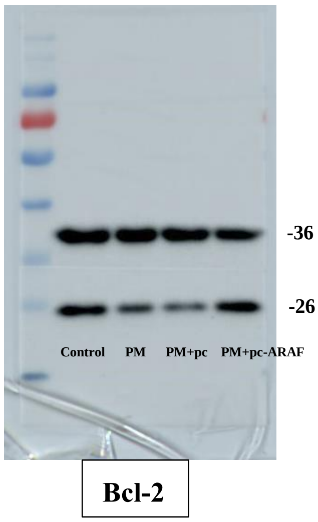

Bcl-2

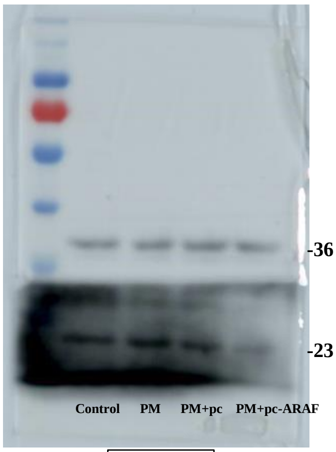

Bax

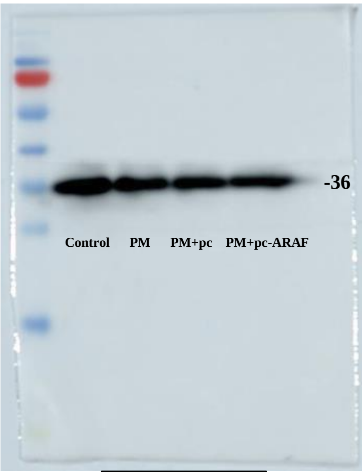

Caspase-3

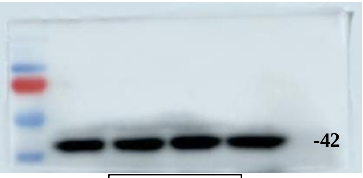

β-actin

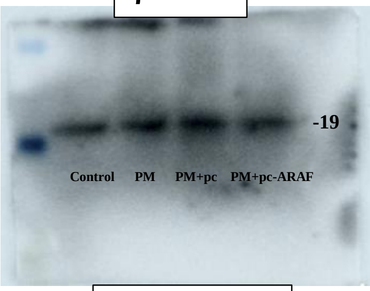

C-Caspase-3
